# Editorial: Cell Fate

**DOI:** 10.3389/fgene.2015.00363

**Published:** 2016-01-11

**Authors:** Laura Buttitta

**Affiliations:** Department of Molecular, Cellular and Developmental Biology, University of MichiganAnn Arbor, MI, USA

**Keywords:** differentiation, gene expression, stem cells, transcription factors, chromatin remodeling, epigenetics

## Introduction

The complexity and plasticity of cell fate determination has intrigued cell and developmental biologists for decades. Cellular differentiation is the acquisition of specialized characteristics; which is intimately associated with changes in gene expression, alterations of chromatin, and changes in nuclear architecture. Differentiating tissues exhibit a progressive restriction of cellular plasticity. However, the regenerative ability of some organisms has revealed an amazing capacity for dramatic switches in cell fate, through trans-differentiation and de-differentiation (Sánchez Alvarado and Tsonis, 2006). Furthermore, the groundbreaking work on somatic cell nuclear reprogramming and induced pluripotency has revealed that commitment to cell fate can be far more flexible than previously thought (Lensch and Mummery, 2013).

In this research topic on cell fate we aimed to highlight new developments and outstanding questions in our understanding of how chromatin dynamics impact cell fate and cellular reprogramming. We include articles discussing cell fate decisions in a wide variety of contexts and model organisms. The contributions to this topic include review articles, mini-reviews, original research, and perspectives. The work described here encompasses organisms ranging from *C. elegans* to humans and deals with global cell fate issues of sex determination (Lau and Csankovszki), lineage choice (Chin), preventing premature differentiation (Foret et al.) cell fate and cell cycle regulation (Oyama et al.; Julian and Blais; Ma et al.; Parker), nuclear architecture (Talamas and Capelson) and how dynamic transcriptional repressors promote cell fate choices (Kok and Arnosti). We thank the authors, reviewers and editors for contributing to the stimulating discussion of the open questions in this rapidly changing field.

## The plasticity of cell fate

Despite the seemingly irreversible nature of cell fate decisions made during embryonic development, there is substantial literature on cellular reprogramming. This can include de-differentiation of cells to a naïve state, such as induced pluripotency, or it can mean direct reprogramming of cells between different fates. In a mini-review on reprogramming cell fate (Chin), Michael T. Chin summarizes advances made in the direct reprogramming of adult, differentiated cells from one cell fate to another, with a discussion of the impact of this research on strategies for regenerative medicine.

Terminally differentiated and postmitotic cells are at the opposite end of the spectrum from reprogramming in cell fate plasticity. How are cell fates properly maintained in the long-term in postmitotic tissues? In a review, Robb MacLellan and colleagues (Oyama et al.) discuss the specialized cell type of cardiac muscle, which undergoes a transition to a permanently postmitotic state coupled with terminal differentiation. They discuss recent work revealing a network of chromatin-associated factors that cooperate with tumor suppressors such as the Retinoblastoma protein to stably repress cell cycle genes and maintain the postmitotic state. How terminal differentiation and the repressive networks are coordinated remains to be deciphered, but whether they may be safely uncoupled is a question with huge potential impact on cardiovascular therapeutics and regeneration.

The proper maintenance of stem cells in aging tissues is a critical issue underlying age-related tissue decline. Maura Parker examines this issue in a review (Parker) on how signaling and epigenetic changes occur with age in satellite cells, the stem cells for skeletal muscle. She suggests that modulations of chromatin and the epigenetic memory of aging stem cells may be key to therapies aimed at “resetting the aging clock.”

## Nuclear architecture, the cell cycle, and cell fate

Sexual determination occurs by a chromosome-based method in many organisms, which leads to an imbalance in gene dosage between the sexes. Dosage compensation acts to equalize X-linked gene expression between the sexes. In *Caenorhabditis elegans*, dosage compensation is achieved by a complex similar to the mitotic condensin complexes. Alyssa C. Lau and Györgyi Csankovszki discuss in a mini-review how dosage compensation in *C. elegans* shares features with condensed mitotic chromosomes (Lau and Csankovszki), and describe why examining condensins in dosage compensation provides unique insights into the relationship of chromatin compaction during interphase and modulation of gene expression.

There is detailed feedback between chromatin architecture, cell fate decisions and cell cycle regulators, as all three influence each other. We continue the theme of exploring chromatin changes associated with the cell cycle, and discuss directly how the mitotic cell cycle impacts chromatin architecture and cell fate (Ma et al.). We summarize new work in cellular reprogramming and nuclear transfer that addresses a provocative question; is there a cell cycling state or cell cycle phase that can increase cellular plasticity?

The discussion of nuclear architecture and cell fate continues in a review by Jessica Talamas and Maya Capelson, which discusses the nuclear envelope and genome interactions in cell fate decisions (Talamas and Capelson). This review describes the interconnected roles of nuclear compartments and asks whether nuclear envelope composition may serve as an unappreciated “cellular code” for directing cell type-specific gene expression programs through contacts with chromatin.

In a more specific focus on cell cycle regulators (Julian and Blais), Lisa M. Julian and Alexandre Blais discuss the transcription factor family, E2F, best known for its roles in regulating cell cycle genes with its repressive partners, the retinoblastoma family. However here, roles for the E2F family outside of the cell cycle are discussed. These are evolutionarily conserved functions in stem cell fate control in a number of lineages, that reveal pivotal roles for E2Fs in the execution of cell type-specific gene regulatory programs.

## Technical advances in deciphering cell fate regulation

Original research by Chin-Hsing Annie Lin and colleagues describes a new technique for profiling chromatin marks and gene expression in specific cell types (Foret et al.). By exploring the adult neurogenic niche in the brain of a non-human primate, they reveal an enrichment of a repressive chromatin mark, suggesting transcriptional silencing protects against improper lineage differentiation in this critical zone.

Closing with the theme of transcriptional repression, in a Perspective piece Kurtulus Kok and David N. Arnosti ponder how repressive complexes on chromatin can display dynamic associations, leading to cycling expression of target genes (Kok and Arnosti). In several developmental contexts cyclic gene expression can impact cell fate decisions, and oscillations in gene expression are likely to be pervasive. Thus the oscillatory behavior and dynamic association of factors with chromatin will need to be considered more fully if we are to understand cell fate decisions.

## Funding

Work in the Buttitta Lab is supported by the NIH (GM086517 and AG047931) and the University of Michigan Biological Science Scholars Program (BSSP).

### Conflict of interest statement

The author declares that the research was conducted in the absence of any commercial or financial relationships that could be construed as a potential conflict of interest.
